# The practice of key essential nutrition actions among pregnant women in southwest Ethiopia: implications for optimal pregnancy outcomes

**DOI:** 10.1186/s12884-024-06354-w

**Published:** 2024-02-23

**Authors:** Shamil Mudasir, Ebrahim Muktar, Abdu Oumer

**Affiliations:** 1 College of Medicine and Health Sciences, Wolkite University, Welkite, Ethiopia; 2 School of Public Health, College of Medicine and Health Sciences, Haramaya University, Harar, Ethiopia

**Keywords:** Essential nutrition action, Antenatal care, Women nutrition, Ethiopia

## Abstract

**Background:**

Nutrition during pregnancy is a major determinant of human health and child development, and the role of promoting essential nutrition actions (ENA) is of a paramount importance for the health of the mother and newborn. However, the practice of ENA could be hampered by many factors, which need to be understood for tailored actions. This study assessed the practice of key ENAs and associated factors among pregnant mothers in southwest Ethiopia.

**Method:**

A community-based cross-sectional study was employed among 373 pregnant mothers. A simple random sampling method was used to select the study participants. The data was entered into EpiData Manager and exported to SPSS version 21 for analysis. A bivariable logistic regression was conducted to explore the association between independent variables and the outcome variable. Variables with p-values less than 0.25 during bivariable analysis were entered into a multivariable logistic regression model. Level of statistical significance was declared at a p-value below 0.05. The crude and adjusted odds ratios, along with the 95% CI, were estimated to measure the strength of the association between the dependent variables and independent variables.

**Result:**

In this study, 373 pregnant mothers have participated, with a response rate of 97%. A total of 275 (73.7%; 95% CI: 68.9–78.0) women practiced key essential nutrition actions at optimal level. Monthly household income of 2500 ETB (AOR = 0.45, 95% CI: 0.23, 0.89), rural residence (AOR = 2.31, 95% CI: 1.25, 4.4), and poor knowledge of key ENA messages (AOR = 3.36, 95% CI: 1.81, 6.26) were factors that were significantly associated with poor practice of key ENA messages.

**Conclusions:**

The practice of key ENA messages was poor and closely linked to household income, residence, and knowledge of pregnant women’s on ENA key messages. Therefore, nutritional intervention with a focus on intensified nutritional counseling is needed for better adoption of key ENA practices.

## Introduction

Improved nutrition is important at all stages of life, especially during critical periods of life [1]. Among these, the first 1000 days of life, including pregnancy and childhood, are crucial to promoting optimal nutrition and health outcomes [2]. Adequate nutritional status of women is important for good health and increased work capacity of women themselves as well as for the health of their offspring [3]. Adequate maternal nutrition and weight gain during pregnancy are the cornerstones of health for women and their children, affecting pregnancy outcomes [4]. Maternal malnutrition is not only predict early child outcome it also increase risk of non-communicable disease in adulthood [2, 5]. Hence, the energy (340–452 kcal per day) and micronutrient requirements are increased during pregnancy, which allows for adequate weight gain [6].

Currently, the COVID-19 pandemic leads to poor affordability of healthy and nutrient-adequate diets in low- and middle-income countries [7]. By 2022, an additional 9.3 million wasted children and 2.6 million stunted children, 168,000 additional child deaths, 2.1 million maternal anemia cases, 2.1 million children born to women with a low body mass index (BMI), and US$29.7 billion in future productivity losses will have occurred [8]. This emphasizes the need to understand the practice of ENA and its importance in improving women’s nutrition. In 2020, approximately 287,000 women died globally during pregnancy or childbirth, with about 70% of these deaths occurring in sub-Saharan Africa. Additionally, every day, around 6,400 newborns die within their first month of life, resulting in an estimated 2.3 million newborn deaths worldwide in 2021 [9]. Globally, an estimated 2.4 million children died in the first month of life as of 2020 (47% of all child deaths) and the major burden lies in sub-Saharan Africa. This is mainly associated with chronic energy deficiency, poor weight gain in pregnancy, anemia, and other micronutrient deficiencies [10].

About 27% of all births in low and middle-income countries (LMICs) are small for gestational age [11]. Hence, the study revealed that pregnant women had a significantly higher prevalence of insufficient intakes of carbohydrates, proteins, vitamin B1, B2, B3, C, and iron [12] than men. In Ethiopia, maternal malnutrition affects about 30.3% [13], 21.8% [14], and 43.1% [15]. Only about 60% of women took iron folic acid (IFA) tablets during pregnancy, yet 11% of them had good adherence to IFA [16]. Improving dietary adequacy during pregnancy is important to help women accommodate their nutritional requirements [17].

To address this, the Essential Nutrition Action (ENA), composed of the seven key maternal and child nutrition interventions, is key to reducing maternal malnutrition [18]. ENA is a set of highly affordable and effective nutrition intervention approaches that are delivered at health facilities and communities to improve the nutritional status of women and children [19]. These behaviors are exclusive breastfeeding, complementary feeding, nutritional care of sick children, nutrition for women during pregnancy and lactation, prevention of vitamin A deficiency, prevention of anemia, and prevention of iodine deficiency, which can be implemented during pregnancy and from the first period onward [20].

The landmark Lancet Series indicated that implementation of such an intervention could reduce nutrition-related mortality and disease burden by 25% [21]. However, the implementation could be challenged by a lack of training, supervision, and coordination [22]. The use of multiple micronutrient supplements could significantly decrease the risk of low birth weight [23]. Nutrients such as vitamins A, B-6, B-12, folic acid, and zinc also affect embryogenesis, which occurs early in pregnancy and may be related to pregnancy loss and fetal malformations [24]. WHO recommends that pregnant women take daily oral iron and folic acid supplementation (IFAS) and mineral fortified food with iron and iodized salt intake to prevent iron and iodine deficiencies [19]. The government of Ethiopia is implementing ENA in an integrated manner, targeting pregnant women and children under two years of age as essential components of child survival strategies [22].

According to a study in northwestern Ethiopia, only 40% had good dietary practices [25]. Limited evidence is available from studies conducted in Ethiopia, where 51% [26] and 28.7% [27] of pregnant women had optimal ENA practice. In addition, the study conducted in northeast Ethiopia (Woreilu district), showed that 66.4% of the mothers have good knowledge and 68.9% have a good attitude towards key ENA messages [28]. The consumption of a diversified diet is very limited (37.1%) [29], with less frequent meals, and about 20.6% had one additional meal during pregnancy [30]. Furthermore, food taboos are additional challenges limiting food intake [31], where very nutritious foods are usually ignored [32]. For instance, in Ethiopia, 18.2-68% of pregnant women [33] were avoiding at least one food during their pregnancy.

Although the ENA has been implemented since 2005, the levels of implementation and its challenges change over time, and such evidence is scarce. Still, suboptimal practices are prevailing, which could be attributed to many factors where substantial reductions in maternal malnutrition and consequent child malnutrition were not achieved. Therefore, this study was to explore the practice of key ENAs and its determinants among pregnant women in Worabe town, southwest Ethiopia to generate evidence for program planning and intervention.

## Method and materials

### Study setting, design, and period

The study was conducted in Worabe town administration, southern Ethiopia. It is located 177 km away from Addis Ababa, the capital city of Ethiopia. The town administration is organized by three urban and eight rural kebeles (the smallest administrative unit in Ethiopia). According to the 2022 town administration report, the total population size is estimated at 79,408. The reproductive age groups 15–49 years are estimated to be 15,754, and according to the town administrative health office report, the total number of pregnant women accounts for around 2686, and of these, 2632 (98%) had at least one ANC visit. The institutional delivery rate in the town is 95%. In the town administration, there are two governmental health centers and two health posts, and there are also eight private medium clinics that provide maternal and reproductive health services [34]. This study was conducted in Worabe town administration from May 12, 2022, to May 30, 2022.

### Populations and eligibility

All pregnant women who resided in the Worabe town administration during the study period were the source population. All randomly selected pregnant women who lived in the selected Kebeles during the study period were the study population. Pregnant women who are permanent residents (women who live in the study area for more than six months) of the Worabe Town administration of the selected clusters of the kebeles and pregnant women who are between the ages of 15 and 49 years were included in the study. Pregnant women who are mentally ill, have chronic diseases, or are unable to speak or hear were excluded.

### Sample size determination and sampling

**Fig. 1:**
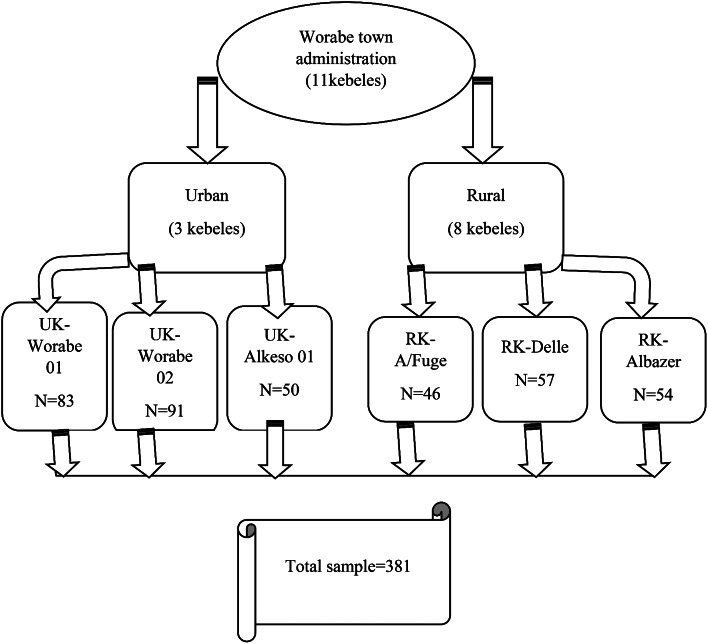
Schematic presentation of sampling procedure (N = sample size, UK = Urban kebeles and RK = Rural kebeles)

The sample size for the first objective was calculated using the single population proportion formula with the following assumptions using the assumption that 20% of the pregnant mothers had optimal nutritional practices during pregnancy [35] with a confidence level of 95%, a marginal error of 5%, and 10% for non-response rate, the sample size became 271 considering non-response. Sample size was determined using a double population proportion formula with the following assumptions; level of confidence to be 95% CI, 5% margin of error, 80% power, and ratio of exposed to unexposed ratio to be 1 using Epi-info version 7. Considering food restriction [36] and dietary knowledge from previous study [25], the sample size was 346 and 96. Finally, we took the larger sample of 381 considering the 10% non-response rate. A simple random sampling method was employed to select the Kebeles and study participants using probability proportional to size allocation (PPS). The total number of kebele in the town administration was stratified into rural and urban areas. Then, 6 kebeles (3 urban and 3 rural) from the town administration was selected randomly from the existing 11 Keble’s (3 urban and 8 rural) randomly, then the sample size was allocated based on Probability Proportional to Size (PPS) sampling technique. The households in the selected Kebeles with pregnant women were identified through house-to-house visits by the data collectors with the guide of Health Extension Workers (HEWs). The data collectors were graduated health professionals. A sampling frame was prepared by registering all the identified eligible pregnant women in each kebeles. After that, simple random sampling was employed to select the required number of pregnant women (Fig. 1).

### Study variables

The dependent variable of the study was the practice of key ENA by pregnant women, dichotomized into good and poor practices. The independent factors considered were socio-demographic and economic characteristics (educational status of mother, residence, marital status, age of mother, occupation, income, family size), behavioral and nutritional information (knowledge and attitude on ENA, health provider advice, source of information, and availability of health facility), maternal characteristics (gravidity, ANC visits, age of pregnancy, and birth spacing), diet and supplements (dietary diversity, micronutrient intake, and IFA).

### Data collection methods and procedures

Pretested and structured questionnaires were adapted from different studies consistent with the conceptual framework and reviewed literature [19, 37–40]. It was prepared in English and translated into Amharic, then again translated back to English to check the consistency. The components of the questionnaires were socio-demographic characteristics, maternal characteristics, maternal ENA knowledge, attitude, and practice, and the dietary diversity adequacy of pregnant women. However, the data were collected by trained health extension workers.

### Measurements of knowledge, attitude and practice towards ENA

ENA is an operational framework for managing the advocacy, planning, and delivery of an integrated package of preventive nutritional actions encompassing exclusive breastfeeding, complementary feeding, nutritional care of sick children, nutrition for women during pregnancy and lactation, prevention of vitamin A deficiency, prevention of anemia, and prevention of iodine deficiency, which can be implemented during pregnancy and periods onward [20]. ENA is a comprehensive package of preventive nutrition measures that consists of seven core components. These components are exclusive breastfeeding, complementary feeding, sick child feeding, nutrition for women during pregnancy and breastfeeding, prevention of vitamin A deficiency, prevention of anemia, and prevention of iodine deficiency [5, 7, 28]. To assess the practice of ENA, a set of questions were used for each relevant component for pregnant women and the total score was calculated. Hence, these items served as indicators to evaluate the extent to which individuals followed the recommended practices of ENA for pregnant women. By utilizing these components and assessment items, ENA aims to promote optimal nutrition and improve health outcomes particularly for pregnant women was the focus of this study. However, for this research, the practice of ENA was based on nutrition for women during pregnancy, with a special focus on the prevention of vitamin A deficiency, anemia, and iodine deficiency. Thus, optimal ENA practice is defined as pregnant women who scored the mean and above mean scores of key ENA practice questions, which were scored out of nine ENA practice items [38].

The knowledge and attitude of women towards ENA were assessed using a set of questions. The knowledge scores were out of 39, and the attitude scores were out of 18. Good knowledge and favorable attitudes of pregnant mothers about key ENA were defined as when the average score is at least the mean of the key ENA knowledge and attitude questions, respectively [37]. The dietary diversity score was composed using ten food groups (minimum dietary diversity score for women) validated by the Food and Agriculture Organization (FAO) in the past 24 h. It is created by summing up the number of food groups consumed over a 24-hour period after coding the food consumption as “1” and “0” otherwise. Each group was assigned a score of “1” if consumed and “0” if not consumed. Then, the scores were summed up for food groups consumed and classified into inadequate dietary diversity when pregnant women consume less than or equal to four food groups and adequate dietary diversity when pregnant women consume five or more food groups out of ten food groups [39].

### Data quality assurance

To ensure the quality of the data, two days of training were provided to data collectors and supervisors. In order to assess the appropriateness of the wording, clarity of the questions, and respondent reaction to the questions and interviewer, a pre-test was conducted on 5% of the calculated sample size of mothers at nearly similar socio-demographic levels in the Kibet town administration. Regular supervision was given during data collection by supervisors and the investigator. An adjustment was made based on the results of the pre-test. Cronbach’s alpha value of > 0.7 was taken to assess the internal consistency (reliability), especially for maternal knowledge, attitude, and practice on ENA and related questions. During the data collection time, close supervision and monitoring were carried out by supervisors and the principal investigator to ensure the quality of the data. The collected data were manually checked for completeness, consistency, and clarity on a daily basis.

### Data analysis

The data were coded and entered into Epi Data version 3.1 and exported for further analysis to SPSS version 21. A descriptive analysis, such as proportions, frequency distribution, and measures of central tendency, was used. Continuous variables like age, family size, and monthly income were first transformed into categorical variables before they were analyzed. The frequencies of all variables in the questionnaires were determined. A bivariable logistic regression was conducted to explore the association between each dependent variable and the outcome variable. Variables with p-values less than 0.25 during bivariable analysis were entered into a multivariate logistic regression model. To measure the strength of the association, a level of statistical significance was declared at a p-value of 0.05. Multivariable logistic regression analysis was used to control for all possible confounders and identify factors associated with ENA practices. The odds ratio and 95% CI were estimated to measure the strength of the association between the dependent variables and independent variables. Model fitness was tested by using the Hosmer-Lemeshow goodness-of-fit and omnibus tests of model coefficients with an enter regression model. By using the variance inflation factor (VIF) test, the tolerance test, and the values of the standard error, the explanatory variables were tested for multi-collinearity before being entered into the multivariable model.

### Ethical approval

Ethical approval was obtained from the institutional Ethical Review Board of Wolkite University, and it was offered to the Worabe town health office. A formal letter of cooperation was prepared from Wolkite University College of Medicine and Health Sciences to the Worabe town health office for further processing to precede the study on selected health institutions. Informed assent was obtained from all the participants. Legally Authorized Representatives of illiterate participants provided informed consent for the study. Only those who were well informed and signed written consent participated in the study, and confidentiality of responses was maintained throughout the research process by giving a code to participants. Informed assent from underage participants and informed consent from their guardians was obtained. All COVID-19 prevention and control measures for were practiced during the data collection procedure.

## Results

### Socio-demographic characteristics

In this study, a total of 373 pregnant women were participated with the cumulative response rate of 98%. The mean ± Standard deviation (SD) of the respondents age was 30.1 ± 6.0 years and the majority 128 (34.3%) of them were aged between 25 and 29 years of age. Majority of the respondents, 320 (85.8%) were Muslim religion followers and 342 (91.7%) were married. Around one thirds of the participants 90(24.1%) were able to read and write as shown in (Table 1).

**Table 1:** Socio-demographic characteristics of pregnant women in Worabe town, southern Ethiopia

Variables	Category	Frequency(n)	Percentage (%)
Age in years	< 20	21	5.6
	21–24	32	8.6
	25–29	128	34.3
	30–34	105	28.2
	≥ 35	87	23.3
Religion	Muslim	320	85.8
	Orthodox	36	9.7
	Protestant	6	1.6
	Catholic	11	2.9
Marital status	Single	21	5.6
	Married	333	89.3
	Separated	6	1.6
	Divorced	5	1.3
	Widowed	8	2.1
Educational status of the mother	Cannot read and write	80	21.4
	Can Read and write	90	24.1
	Primary	69	18.5
	Secondary	73	19.6
	Higher education	61	16.4

Two hundred and nineteen (58.7%) of pregnant women resides in urban areas. Regarding economic status, the majority of pregnant mothers’ monthly household income was between 2500–5000 ETB. The majority of the respondents’ 361 (96.8%) households were headed by husbands. Regarding gestational age, 235 (63%) of pregnant women were between 14 and 28 weeks. More than half (55.8%) had a birth space between 3–5 years (Table 2).

**Table 2:** Socio-demographic characteristics of pregnant women in Worabe town, southern Ethiopia (n = 373)

Variables	Category	Frequency(n)	Percentage (%)
Educational status of the father	Cannot read and write	69	18.5
	Can Read and write	79	21.2
	Primary	58	15.5
	Secondary	63	16.9
	Higher education	104	27.9
Occupation of the mother	Non-employed	190	50.9
	Housewife	108	29.0
	Employed in government institution	67	18.0
	Merchant	8	2.1
Residence	Urban	220	59.0
	Rural	153	41.0
Monthly household income in ETB	< 2500	125	33.5
	2500–5000	160	42.9
	> 5000	88	23.6
Number of family member	< 4	78	20.9
	5–6	139	37.3
	7–8	138	37.0
	> 9	18	4.8
Parity	Primipara	34	9.1
	Multipara	339	90.9
Gestational age	< 14weeks	5	1.3
	14-28weeks	235	63.0
	> 28weeks	133	35.7
Inter pregnancy interval	< 3 years	150	40.2
	3–5 years	208	55.8
	> 5 years	15	4.0
Head of household	Husband	361	96.8
	Myself	12	3.2
ANC follow up	Yes	278	74.5
	No	95	25.5

### Knowledge, attitude and practice of key ENA message

**Fig. 2:**
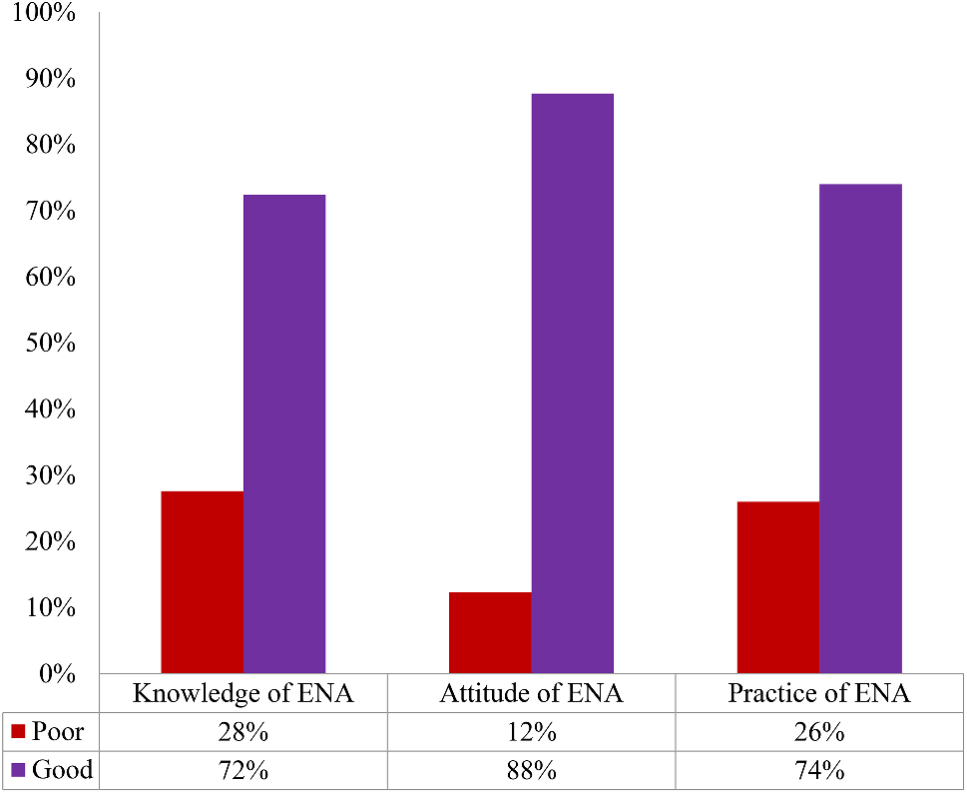
Knowledge, attitude and practice of key ENA among pregnant women in Worabe town, Ethiopia

Almost three-fourths (270%) of pregnant women had good knowledge of key ENA messages. Similarly, 88% and 74% had favorable attitudes and good practices towards ENA messages for pregnant women (Fig. 2).

Out of 373 pregnant women, 260 (70%) consumed adequate food groups. The mean standard deviation (SD) of the dietary diversity score is 5.321.42. Regarding the food groups consumed by pregnant women, around 355 (95.2%) reported to consume grains, white roots and tubers, and plantains. Moreover, more than three-fourth of pregnant women (280 (75.1%)) did not consume meat, poultry, or fish in the previous 24-hours preceding the survey (Table 3).

**Table 3:** Individual food group consumption by pregnant women in the previous 24 h in Worabe town, Ethiopia

Variables	Consumed/not consumed	Frequency(n)	Percent (%)
Grains, white roots and tubers, and plantains	Not consumed	18	4.8
	Consumed	355	95.2
Pulses (beans, peas and lentils	Not consumed	118	31.6
	Consumed	255	68.4
Nuts and seeds	Not consumed	254	68.1
	Consumed	119	31.9
Dairy	Not consumed	227	60.9
	Consumed	146	39.1
Meat, poultry and fish	Not consumed	280	75.1
	Consumed	93	24.9
Eggs	Not consumed	210	56.3
	Consumed	163	43.7
Dark green leafy vegetables	Not consumed	134	35.9
	Consumed	239	64.1
Other vitamin A-rich fruits and vegetables	Not consumed	186	49.9
	Consumed	187	50.1
Other vegetables	Not consumed	111	29.8
	Consumed	262	70.2
Other fruits	Not consumed	209	56.0
	Consumed	164	44.0

More than a fourth of pregnant women (275, 73.7%; 95% CI, 68.9–78%) practiced the key ENA practice at an optimal level. whereas 98 (26.3%: 95% CI; 22.0–31.1%) of the respondents do not practice key ENA. Regarding additional meals, more than half of pregnant women (220; 59%) ate one additional meal every day, and 232 (62.2%) added iodized salts at the end of cooking food (Table 4).

**Table 4:** Practice of practice of key ENA message among pregnant women in Worabe town, SNNPR, Ethiopia, 2022 (n = 373)

Variables	Categories	Frequency (*n* = 373)	Percent (%)
Did you eat one additional meal every day?	No	153	41.0
	Yes	220	59.0
Did you eat a variety of foods, particularly animal products plus fruits and vegetables during pregnancy?	No	126	33.8
	Yes	247	66.2
During the day and night, did you eat any of the following foods; Fruits-ripe mango, ripe papaya, cantaloupe, apricot	No	246	66.0
	Yes	127	34.0
Did you receive iron–folic acid?	No	130	34.9
	Yes	243	65.1
Did you eat meat (liver, kidney and heart) and animal product?	No	120	32.2
	Yes	253	67.8
Did you eat green leafy vegetables during pregnancy?	No	84	22.5
	Yes	289	77.5
What kind of salt did you use while you cook family food?	None-iodized	104	27.9
	Iodized	269	72.1
When did you add salt in to the stew?	At the end	232	62.2
	At the middle	107	28.7
	At the beginning	34	9.1
Did you store salt in dark closed container?	No	70	18.8
	Yes	303	81.2

### Factors affecting practice of key essential nutrition action

The association between the dependent and independent variables was explored by both bivariable and multivariable binary logistic regression. The bivariable logistic regression analysis shows that age of women in years, educational status of the mother, educational status of the father, occupation of the mother, monthly household income, residence, parity, number of family members, source of health and nutrition information, inter-birth interval, attitude toward key ENA messages, and knowledge of key ENA messages were the factors associated with the practice of key ENA messages at a p-value of 0.25. Then, in the multivariable logistic regression analysis, some variables, such as monthly household income, residence, and knowledge of key ENA messages, were significantly associated with the practice of key ENA messages at a p-value of 0.05. Pregnant women whose average monthly household income was 2500 ETB were 0.45 (AOR = 2.22; 95% CI: 1.12–4.35) times less likely to practice key ENA messages than those whose average monthly household income was > 5000 ETB. Pregnant women living in rural areas were 2.3 times more likely than those living in urban areas to practice poor key ENA messages (AOR = 2.31, 95% CI: 1.25, 4.4). Furthermore, pregnant women who had poor knowledge of key ENA messages were three times more likely to practice key ENA messages poorly than pregnant women who had good knowledge of key ENA messages (AOR = 3.36; 95% CI: 1.81–6.26) (Table 5).

**Table 5:** Bivariable and multivariable analysis of factors associated to key ENA practice among pregnant women in Worabe town, SNNPR, Ethiopia

Variables	Key ENA Practice		COR (95%CI)	AOR (95%CI)	P-value
	Suboptimal	Optimal			
**Age of women in years**					
< 20	8(8.2%)	13(4.7%)	0.479(0.16,1.47)	2.08(0.76–11.1)	0.120
21–24	18(18.4%)	14(5.1%)	1.846(0.7,4.8)	1.67(0.49,5.7)	0.413
25–29	32(32.7%)	96(34.9%)	2.785(1.01,7.66)	3.17(0.79,12.69)	0.102
30–34	19(19.4%)	86(31.3%)	1.934(0.71,5.3)	1.81(0.44,7.45)	0.414
≥ 35	21(21.4%)	66(24%)	1	1	
**Monthly household income in ETB**					
< 2500	33(33.7%)	92(33.5%)	1.24(0.73–2.08)	2.22(1.12–4.35)	0.022
2500–5000	49(50%)	111(40.4%)	1.61(0.32–1.21)	1.43(0.56–3.23)	0.504
> 5000	16(16.3%)	72(26.2%)	1	1	
Parity					
Primipara	15(15.3%)	19(6.9%)	2.43(1.18,5.0)	1.10(0.36–3.33)	0.877
Multipara	83(84.7%)	256(93.1%)	1	1	
**Attitude of key ENA message**					
Poor	16(16.3%)	30(10.9%)	1.59(0.83,3.07)	1.83(0.82,4.1)	0.140
Good	82(83.7%)	245(89.1%)	1	1	
**Knowledge of key ENA message**					
Poor	47(48.0%)	56(20.4%)	3.6(2.2,5.9)	3.36(1.81,6.26)	< 0.0001
Good	51(52.0%)	219(79.6%)	1	1	
**Residence**					
Urban	47(48%)	173(62.9%)	1	1	
Rural	51(52%)	102(37.1%)	1.84(1.16,2.93)	2.31(1.25,4.4)	0.011
**Number of family member**					
< 4	32(32.7%)	46(16.7%)	2.07(1.14,3.74)	1.03(0.43,2.48	0.941
5–6	35(35.7%)	104(37.8)	2.50(1.37,4.59)	1.41(0.49,4.01)	0.525
7–8	30(30.6%)	108(39.3%)	11.8(1.5,93.4)	5.57(0.52,59)	0.155
> 9	1(1%)	17(6.2%)	1	1	

*Note* Hosmer and Lemeshow’s Test was 0.676 depicting model fitness

Additionally, primipara and pregnant women with poor attitudes (AOR = 1.83; 95% CI: 0.82–4.1) had 10% and 83% lower odds of practicing key ENA messages, respectively. Those women with poor knowledge of ENA had 3.36 times increased odds of having poor ENA practices (AOR = 3.36; 95% CI: 1.81–6.26). Those with extended family and from rural areas (AO = 2.31; 95% CI: 1.25–4.4) are more likely to practice key ENA messages poorly (Table 5).

## Discussion

This study assessed the practice of key ENA and associated factors among pregnant mothers. Accordingly, 73.7% (95% CI; 68.9–78%) of pregnant women had optimal ENA practice. This study’s finding is higher than in the previous study done in southwest Ethiopia, where 28.7% [27] and in northeast Ethiopia, 46.5% [38]. The possible difference might be due to a difference in study season, and increased in maternal and child health service access, and increase maternal awareness about key ENA messages. Another study also showed that 60.7% of pregnant women reported poor dietary practices [25] which could affect access to diversified foods associated with seasonal variations [41, 42]. Another study from Turkey also reported that the practice good breast feeding was 40.8% where 59.8% exclusively fed their child [43]. This is mainly due to variation in the accessibility of vitamin and mineral rich fruits and vegetables limiting their practice and reducing the diet diversity. This could be further threatened by the rising price of nutritious foods associated with inflation [44] in addition to the huge knowledge gap in proper feeding practices [28].

Average monthly household income is significantly associated with the practice of key ENA, as pregnant women whose average monthly household income was 2500 ETB (AOR = 2.22; 1.12–4.35) had a higher odd of having suboptimal ENA practice compared to wealthier families. This is supported by the previous study done in Northeast Ethiopia [38], Northwestern Ethiopia, where household income is negatively associated with dietary practice (25), One possible explanation for this might be that when a woman had a high household monthly income, she might buy luxury food commodities such as canned food at supermarkets. And also, she might not accept nutritional counseling from health professionals. This leads to malpractice in key essential nutrition actions.

Furthermore, this study noted that pregnant women whose residence was rural were 2.3 times more likely to practice key ENA messages poorly than urban dwellers. Unfortunately, the previous studies that were conducted on key ENA practices among pregnant women did not support this study’s finding [25]. Since the possible justification might be that in Ethiopia, the majority of health facilities are available in urban areas, Due to this, the accessibility and utilization of health facilities in urban women are comparatively higher than in rural women. In addition to this, the accessibility of nutrition and related information among urban dwellers is higher than that of their counterparts. Nutritional behaviors and access to services also greatly vary by residence.

Nutritional knowledge among pregnant women was significantly associated with optimal ENA practice. Pregnant women who had poor knowledge of key ENA messages were three times more likely to have poor practice of key ENA messages compared to their counterparts. This study is in line with the previous study done in America [45]. Similarly, studies in Ethiopia, Northeast Ethiopia [38], Northwest Ethiopia [35] and Guto Gida Woreda, East Wollega Zone, Ethiopia [46] also indicated that women’s dietary knowledge was a predictor of good ENA practices. The possible justification might be that improving pregnant women’s knowledge of key ENA messages is the cornerstone for implementing sustainable strategies to improve appropriate ENA practices. Hence, poor knowledge of maternal nutrition leads to poor dietary intakes and results in undernutrition [14]. It has been also indicated that building good attitude could help to increase ENA practices. Moreover, the local food production patterns, accessibility of foods and the food security status could affect the uptake of key ENA practice [47].

The study is representative due to its community-based cross-sectional design. The study had an adequate sample size. A standard questionnaire developed by multiple publications was employed with only slight adaptation to the local context. As a cross-sectional study in nature, it might have drawbacks based on the actual situation of the seasonal difference in food availability in the study area. The study may introduce social desirability and recall bias. However, the study area might not be representative to all pregnant women in the study area, and limiting its generalizability.

## Conclusion and recommendation

Based on the findings of this study, the practice of key ENA is relatively good, but it needs further improvement. Suboptimal ENA practice was associated with household income, residence, and maternal knowledge, which can be targeted in implementing ENA packages particularly to the pregnant women who reside in rural areas. This study implicates the need for enhanced and targeted implementation of ENA to improve maternal nutrition knowledge and have a good implementation of the key messages delivered. Health professionals should get refresher training on ENA and ANC services for better delivery of ENA key messages.

## Data Availability

All the data generated in this study are within the submitted manuscript and its supporting information files. Further datasets can be shared by corresponding author upon reasonable requests.

## Abbreviations

ANC: Antenatal Care
AOR: Adjusted Odds Ratio
BMI: BMI-Body Mass Index
CI: Confidence Interval
COR: Crude Odds Ratio
ENA: Essential Nutrition Action
ETB: Ethiopian Birr
FAO: Food and Agricultural Organization
HEW: Health Extension worker
IFAS: Iron and Folic Acid Supplementation
SD: Standard Deviation
UNICEF: United Nation Children Education Fund
VIF: Variance Inflation Factor
WHO: World Health Organization
